# SALVAGE ALPPS PROCEDURE FOR FAILED PORTAL VEIN EMBOLIZATION

**DOI:** 10.1590/0102-672020230058e1776

**Published:** 2023-12-04

**Authors:** João Victor Vecchi Ferri, Flávia Heinz Feier, Leandro Armani Scaffaro, Leticia Maffazioli, Celina Pereira Hallal, Cleber Rosito Pinto Kruel, Marcio Fernandes Chedid, Tomaz de Jesus Maria Grezzana

**Affiliations:** 1 Universidade Federal do Rio Grande do Sul, Porto Alegre University Hospital, Hepatobiliary Surgery and Liver Transplantation Unit, Porto Alegre (RS), Brazil.

## INTRODUCTION

Large tumors located in the right liver lobe may preclude the safe performance of right trisectionectomy due to a small left lateral segment remaining. First reported by Makuuchi et al. in 1980, embolization of the right portal vein (PV) is performed to trigger contralateral hypertrophy and enable the performance of right trisectionectomy^6^. The obstruction of the left PV due to inadvertent migration of embolizing agents following right portal vein embolization (PVE), as preparation for resection of large unresectable colorectal liver metastasis (CRLM), may preclude curative liver resection^1^.

The ALPPS (Associating Liver Partition and Portal vein Ligation for Staged hepatectomy) procedure usually induces fast hypertrophy of the left liver remnant^8,10^. Inclusion criteria are patients with extensive bilobar colorectal liver metastases and a predicted future liver remnant <30%. A rare case of utilization of the ALPPS procedure as rescue surgery after a failed PVE is reported herein.

## CASE REPORT

A 54-year-old female presented with a large metachronous CRLM involving right hemiliver and segment IVa, 22 months after a left hemicolectomy. After a 4-cycle oral FOLFIRI chemotherapy, she was evaluated for a right trisectionectomy, but the future liver remnant (FLR=22%) was deemed as insufficient. To induce FLR hypertrophy, right PVE guided by ultrasound was performed through peripheral right PV branches and superselective catheterization of anterior and posterior portal branches employing polyvinyl alcohol particles (ContourT, Boston Scientific, Cork, Ireland®). A mixture of cyanoacrylate glue and lipiodol was injected under fluoroscopic guidance. Portography revealed adequate right PVE.

**Figures 1:**
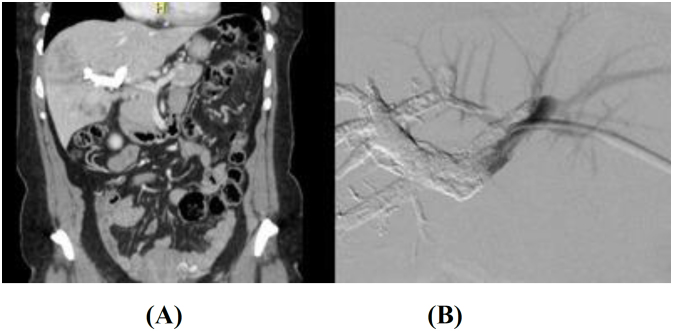
(A) Computed tomography scan revealed thrombosis of left portal vein small branches (B) secondary to migration of embolizing material inside left portal vein.

**Figures 2:**
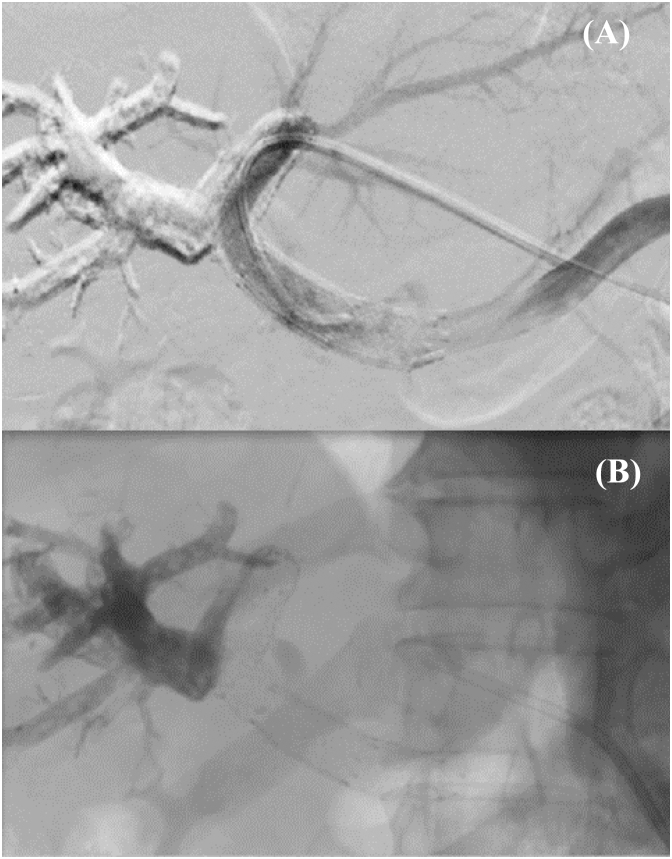
(A) Balloon angioplasty and (B) Stent placement in the left portal vein.

Four weeks later, a computed tomography (CT) scan revealed thrombosis of left PV small branches secondary to migration of embolizing material inside LPV (Figures 1A, 1B), explaining the lack of hypertrophy of the left hemiliver (FLR=25%). Balloon angioplasty and stent placement in the LPV were carried out (Figures 2A, 2B).

**Figures 3:**
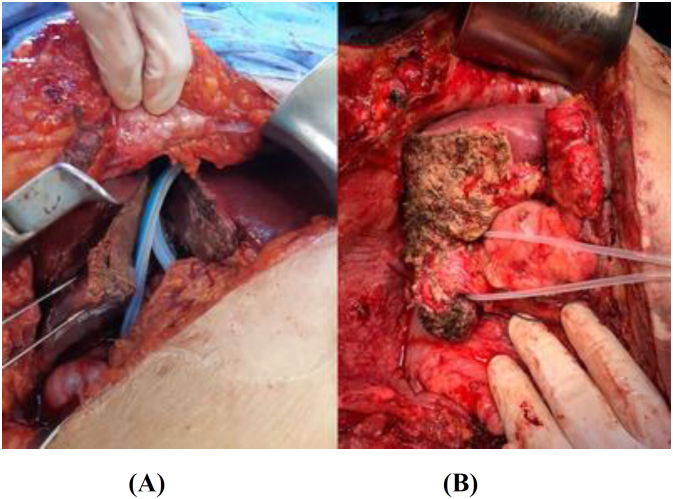
(A) Associating liver partition and (B) Portal vein ligation for staged hepatectomy.

ALPPS^10^ was taken out seven weeks later (Figure 3A) (predicted FLR=29% two weeks thereafter). Then, a right trisectionectomy was operated as ALPPS completion (Figure 3B).

One month later, a CT scan identified a non-occlusive thrombosis compromising the inferior vena cava (from the left hepatic vein to the left common iliac vein). Rivaroxaban was initiated and kept for 6 months, and patient was discharged home 41 days after the hepatectomy. The patient received an 8-cycle FOLFIRI adjuvant scheme. She remains stable, with no signs of recurrent disease or thrombosis 21 months after the procedure. This study was approved by the Institutional Board (number 2017-0271), and the patient signed informed consent for this report.

## DISCUSSION

Complete resection of CRLM is the best treatment to achieve long term survival^3,5^. Resection of adjacent structures may be necessary in order to achieve free tumor margins^2^. In some cases, whatsoever insufficient FLR due to a small left lateral segment may preclude such approach.

PVE is used in order to induce contralateral lobe hypertrophy, aiming to achieve a safe FLR volume. However, such hypertrophy is not always reached. PVE generally triggers a 20 to 40% increase in FLR. With the advent of ALLPS, patients previously considered non-operative candidates are now being able to benefit from a complete tumor resection. Even if the patient experiences a failed or insufficient PVE, “salvage ALPPS” can still be carried out^9^. A higher morbi-mortality should be expected in such cases, though^4^. Due to previous PVE, only liver partition is generally performed without the need for PV ligation.

Rolinger et al. presented a case of inadvertent nontarget portal thrombosis of the FLR following PVE. An ALPPS procedure with concomitant thrombectomy of the left portal vein was used as a rescue strategy for this patient, concluding that it can be used in the event of technical failures or complications following PVE, even in patients with perihilar carcinoma^7^. In the case reported herein, ALPPS was utilized in a scenario of failed PVE. Salvage ALPPS may be a therapeutic option for failed PVE even in the event of inadvertent contralateral migration of embolizing material.
